# Translucency and Strength of Lithium Disilicate for Computer-Aided Design and Manufacturing at Different Thermal Temperatures and Thicknesses: An In Vitro Study

**DOI:** 10.3390/ma17020396

**Published:** 2024-01-12

**Authors:** Chong-Yang Li, Kyung-So Jeong, Jae-Seob Shin, Ji-Suk Shim, Jae-Jun Ryu

**Affiliations:** 1 Department of Medicine, Korea University Graduate School, Seoul 08308, Republic of Korea; lcy114423@gmail.com (C.-Y.L.); kenworld@naver.com (K.-S.J.); js_3538@naver.com (J.-S.S.); 2 Department of Prosthodontics, Korea University Guro Hospital, Seoul 08308, Republic of Korea; 3 Department of Prosthodontics, Korea University Anam Hospital, Seoul 02841, Republic of Korea

**Keywords:** lithium disilicate glass–ceramic, process: translucency, flexural strength, firing temperature, thickness

## Abstract

To manufacture dental restorations composed of lithium disilicate (LD) through the computer-aided design and manufacturing (CAD/CAM) process, thermal refinement is an essential process that can affect the optical and mechanical properties of ceramics. In this study, we aimed to evaluate the translucency and flexural strength of lithium disilicate glass–ceramic for CAD/CAM using different thermal refinement schedules and thicknesses by measuring the total transmission of light through the specimen and calculating the peak load of the specimen until fracture in a piston-on-three-ball test, respectively. The results showed that a lower translucency was exhibited in thicker specimens, and the flexural strength decreased in the order of 1.0, 0.5, and 2.0 mm (*p* < 0.05). The lithium disilicates thermally refined at a heat of 820 degrees were shown to have the highest biaxial flexural strength (*p* < 0.05). These findings suggest that it is possible to adjust transparency and strength according to the clinical situation by choosing an appropriate thickness and thermal refinement process.

## 1. Introduction

Lithium disilicate (LD, Li_2_Si_2_O_5_) glass–ceramic is one of the most popular dental restorative materials because of its high mechanical strength and favorable esthetic properties [1]. Recently, computer-aided design and computer-aided manufacturing (CAD/CAM) systems have been the main techniques for restoration manufacturing of LD glass–ceramic and are less technically sensitive, allowing the fabrication of more detailed and precise restorations and reducing production time and costs [2,3].

Supplemental thermal treatment is necessary to manufacture dental restorations with LD through the CAD/CAM process. Restoration manufacturing of LD glass–ceramics for CAD/CAM involves serial procedures, including industrial casting of blocks, milling, and a final thermal refinement called crystallization [4]. For an efficient milling process, the partially crystallized lithium metasilicate (LM, Li_2_SiO_3_) state is used to increase the cutting efficiency, maximize the life of the milling tools, and make the process easier because of its mild to moderate strength and hardness [5]. Supplemental thermal refinement after milling causes structural changes in the ceramic that destabilize LM and induce the growth and maturation of LD [6,7]. Supplemental thermal treatment can affect the optical and mechanical properties of LD, which are major factors in determining the results of treatment with ceramics in the dental clinic [8]. Translucency is a primary factor in controlling the esthetic outcome of dental ceramics, and the direct transmittance of light has been reported to be a reliable method for evaluating the translucency and opacity of aesthetic restorative materials [9]. Mechanical strength is an important factor that controls the clinical success of dental restorations, which determines the longevity of the restoration [10,11]. Thermal treatment, where the temperature drives the dissolution of LD, causes differences in crystal size, which affect the optical and mechanical properties of ceramics [12].

The thickness of ceramics, which is determined by the amount of tooth reduction, is one of the major factors affecting the optical and mechanical properties of ceramic materials [13]. Previous studies found that the translucency parameter of dental ceramics, including both glass–ceramics and zirconia ceramics, is significantly influenced by thickness and there is a decrease in translucency with an increase in thickness [14]. In addition, it has been reported that the biaxial flexural strength values increased with an increasing core thickness in all the ceramic materials tested, including glass-infiltrated aluminum oxide cores, lithium-disilicate-reinforced glass–ceramic cores, and yttrium-stabilized zirconia cores, regardless of aging [15]. However, a previous study found that increasing the thickness had little effect on the overall flexural strength for two dental ceramic materials analyzed, including Dicor^®^ (a fine-grained glass–ceramic) and Cerestore^®^ (a magnesia–alumina spinel core ceramic). A total of 67% of failed Dicor^®^ crowns and 56% of failed Cerestore^®^ crowns had failure initiation sites located in regions of thickness greater than 1.5 mm [16]. In addition, information about the optical and mechanical properties at different thicknesses is essential for the dental clinician to select the appropriate clinical case for the use of LD and determine the proper amount of tooth reduction. However, studies on the optical properties and the flexural strength of LD glass–ceramic for CAD/CAM at different firing temperatures in the process of thermal refinement and at different thicknesses are very limited in the literature.

Herein, the purpose of this study is to evaluate the translucency and flexural strength of LD glass–ceramics with various thicknesses processed via supplemental thermal treatment at different firing temperatures for CAD/CAM. To achieve this purpose, partially sintered LD specimens with three different thicknesses (0.5, 1.0, and 2.0 mm) were thermally refined at six different firing temperatures (780, 800, 820, 840, and 860 °C). The composition and microstructure of ceramics were analyzed via X-ray diffraction (XRD), and the optical and mechanical properties were evaluated. The null hypotheses were that (1) the translucency and flexural strength of LD glass–ceramics for CAD/CAM are not affected by the temperature of thermal refinement, and (2) the translucency and flexural strength of LD glass–ceramics for CAD/CAM are not affected by the thickness of the ceramic.

## 2. Materials and Methods

### 2.1. Glass Block Preparation

The composition of LD parent glass was 72.6 mol% SiO_2_, 10.7 mol% Al_2_O_3_, 7.9 mol% K_2_O, 2.1 mol% CaO, 0.3 mol% TiO_2_, 4.7 mol% Na_2_O, 1.1 mol% Li_2_O, and 0.5 mol% MgO, which has been reported previously [8]. The glass batch was melted in an electric kiln (Fredrickson Kiln Co., Alfred, NY, USA) at 1316 °C for 7 h, after which the molten glass was cooled to room temperature to form the precursor glass block.

### 2.2. Heat-Treatment Schedule

After a 3 min standby duration at 400 °C, the temperature was raised at a heating rate of 60 °C/min to five different firing temperatures, 780, 800, 820, 840, and 860 °C, with a 15 min holding time using a furnace (Ivoclar Vivadent Programat, Ivoclar Vivadent AG, Schaan, Liechtenstein). Fired glass blocks (Prototype, HASS, Gangneung, Republic of Korea) were quenched in a crucible and heat-treated for inducing nucleation sequentially.

### 2.3. X-ray Diffraction (XRD)

The specimens obtained after heat treatment at the five different firing temperatures were selected and pulverized to detect the crystalline phases of the LD glass–ceramic using a MultiFlex X-ray diffractometer (Rigaku, Tokyo, Japan) under Cu-Kα radiation in the 2θ range from 10° to 60° with a scanning speed of 6°/min.

### 2.4. Translucency Measurement

A total of 75 disc-shaped specimens (diameter of 10 mm) of three different thicknesses (0.5, 1.0, 2.0 mm) were prepared from the partially crystallized blocks by using a cutting machine (Isomet Low-speed, Buehler, Lake Bluff, IL, USA) under constant water cooling. Each specimen underwent a series of surface grinding and polishing processes with wet 600-, 800-, 1000-, and 1200-grit silicon carbide paper (CarbiMet, Buehler, Lake Bluff, IL, USA) in a grinder/polisher machine (EcoMet 30, Buehler, Lake Bluff, IL, USA), and then underwent separate heat treatment at the five different firing temperatures, respectively (5 specimens for each group). After firing, both surfaces of each specimen were wet-finished by using 1200-grit silicon carbide paper in a grinder/polisher machine, and then underwent ultrasonic cleaning with distilled water for 10 min and was dried with compressed air. The definitive thickness was determined using a digital micrometer (Mitutoyo Corp., Tokyo, Japan) with an error of ±0.05 mm. Translucency measurements were taken by measuring the total transmission of light through the specimen with a spectrophotometer (UV-2600; Shimadzu, Kyoto, Japan) at a wavelength between 400 and 700 nm. Each specimen was measured three times and the average percent of total transmission (T%) was calculated by using the following formula: T% = (L_specimen_/L_source_) × 100. L_source_ was recorded with no specimen before each measurement as a baseline value.

### 2.5. Biaxial Flexural Strength

The biaxial flexural strength was measured in a piston-on-three-ball test using a universal testing machine (AGX-KN10, Shimadzu Co., Kyoto, Japan) based on the International Organization for Standardization (ISO) 6872:2015 standard [17]. A total of 225 disc-shaped specimens (diameter of 13 mm) with three different thicknesses (0.5, 1.0, 2.0 mm) were also prepared from the partially crystallized blocks by using the same cutting machine under the same preparation conditions as the specimens in the translucency measurements. These specimens then underwent separate heat treatments at the five different firing temperatures, respectively (15 specimens for each group). After a similar preparation procedure to that of the translucency measurements, including a series of grinding, polishing, and cleaning processes and determining the definitive thickness, each specimen was placed centrally on the three steel balls (diameter of 3 mm) positioned 120° apart on a support circle (diameter of 12 mm). A load was applied with a flat punch-shaped rod (diameter of 1.2 mm) at a crosshead speed of 1 mm/min to the center of the specimen until fracture. The biaxial flexural strength (MPa) was calculated from the peak load of the failure.

### 2.6. Statistical Analysis

Descriptive data for translucency and biaxial flexural strength were presented as means ± standard deviation. The Shapiro–Wilk test was applied for testing the distribution normality of small samples and showed a non-normal distribution (*p*-value less than 0.05). As the study groups were independent, a Kruskal–Wallis test was applied to compare the differences between the study groups, and then multiple comparisons were performed when the overall test showed significant differences across samples. All statistical tests were performed by using a statistical software program (SAS v9.4; SAS Institute, Cary, NC, USA) and a *p*-value less than 0.05 was considered statistically significant.

## 3. Results

### 3.1. XRD Analysis

**Figure 1:**
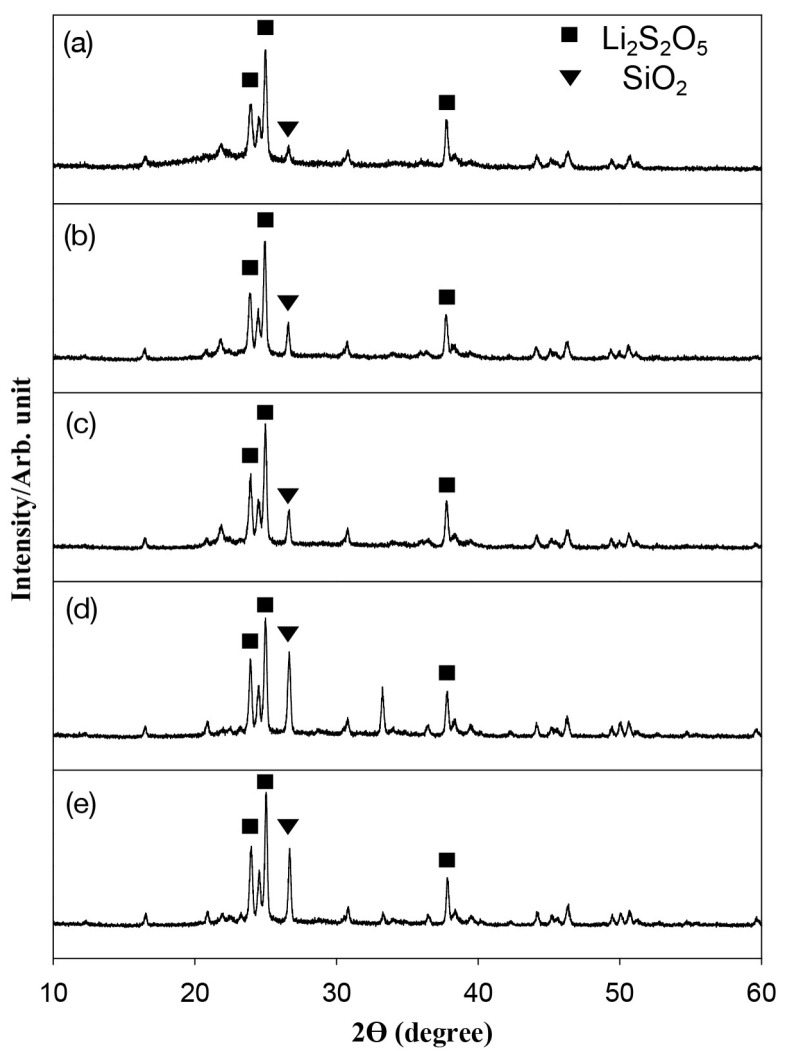
XRD of lithium disilicate glass–ceramics at different firing temperatures: (**a**) 780 °C, (**b**) 800 °C, (**c**) 820 °C, (**d**) 840 °C, and (**e**) 860 °C.

Figure 1 shows the XRD patterns of the LD glass–ceramic for different firing temperatures. The main crystalline phase detected was LD, and the major peaks of the phase were observed at the 2θ values of 23.85, 24.39, and 24.88. The specimens treated at higher firing temperatures exhibited a higher peak of LD. The peaks of SiO_2_ increased with increasing the firing temperature from 780 °C to 840 °C but slightly decreased at 860 °C. Table 1 presents the primary grain size of LD glass–ceramic for different designed firing temperatures, which was calculated via the Scherrer equation. The primary grain size increased as the firing temperature increased, with the smallest value of 28.4 ± 5.8 nm at 780 °C and the largest value of 35.5 ± 4.0 nm at 860 °C.

### 3.2. Translucency

**Figure 2:**
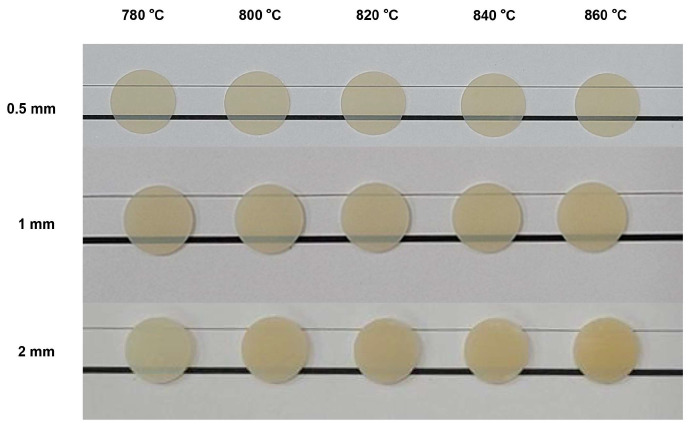
Optical images of lithium disilicate glass–ceramic specimens for different designed firing temperatures and thicknesses.

**Figure 3:**
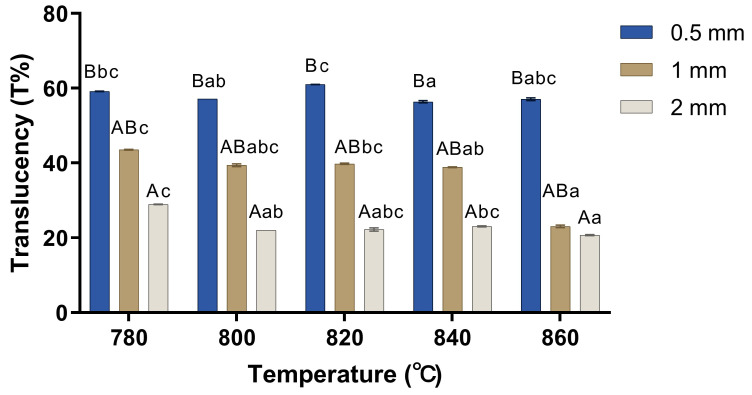
Comparison of translucency (T%) at different designed firing temperatures (°C) and different thicknesses (mm). Similar letters (uppercase for the comparison of translucency of different thicknesses in the same firing temperatures and lowercase for the comparison of translucency of different temperatures in the same thickness) indicate homogenous subsets among experimental groups (*p* > 0.05).

Figure 2 displays the apparent translucency of lithium disilicate glass–ceramic specimens for different firing temperatures and thicknesses. Figure 3 shows the comparison of translucency at different designed firing temperatures and different thicknesses. Regarding the 0.5 mm specimens, there were significant differences in translucency between 780 °C and 840 °C, 800 °C and 820 °C, and 820 °C and 840 °C. The 1 mm specimens exhibited significant differences in translucency between 780 °C and 840 °C, 780 °C and 860 °C, and 820 °C and 860 °C. Regarding the 2 mm specimens, statistical significances were seen between 780 °C and 800 °C, 780 °C and 860 °C, and 840 °C and 860 °C.

When comparing the translucency at different designed thicknesses in each firing temperature group, there was a decrease in the translucency with an increase in thickness. However, for each temperature group, a significant difference was seen only between 0.5 mm and 2 mm (*p* < 0.05).

Table 2 shows the effect of thickness and temperature on translucency. A significantly higher translucency was shown, reducing in the order of 0.5 mm, 1 mm, and 2 mm specimens (*p* < 0.05). There was no significant difference in the translucency values between the designed firing temperature groups (*p* > 0.05).

### 3.3. Biaxial Flexural Strength

**Figure 4:**
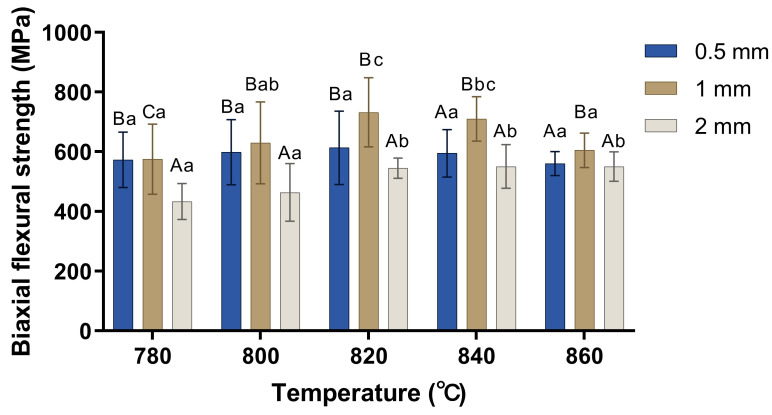
Comparison of biaxial flexural strength (MPa) at different designed firing temperatures (°C) and different thicknesses (mm). Similar letters (uppercase for the comparison of biaxial flexural strength of different thicknesses in the same firing temperatures and lowercase for the comparison of biaxial flexural strength of different temperatures in the same thickness) indicate homogenous subsets among experimental groups (*p* > 0.05).

Figure 4 shows the comparison of the biaxial flexural strength at different firing temperatures and different thicknesses. For the 0.5 mm specimens, there was no significant difference in the biaxial flexural strength between any two temperature groups. Regarding the 1 mm specimens, there were significant differences in the biaxial flexural strength between 780 °C and 820 °C, 780 °C and 840 °C, 800 °C and 820 °C, 820 °C and 860 °C, and 840 °C and 860 °C. The 2 mm specimens exhibited significant differences in biaxial flexural strength between 780 °C and 820 °C, 780 °C and 840 °C, 780 °C and 860 °C, 800 °C and 820 °C, 800 °C and 840 °C, and 800 °C and 860 °C.

For the 780 °C group, a significantly higher biaxial flexural strength was shown that decreased in the order of 1 mm specimens, 0.5 mm specimens, and 2 mm specimens. For both the 800 °C and 820 °C groups, 2 mm specimens exhibited a significantly lower biaxial flexural strength than the other two thickness groups. For both the 840 °C and 860 °C groups, 1 mm specimens showed a significantly higher biaxial flexural strength than the other two thickness groups.

Table 3 shows the effect of thickness and temperature on the biaxial flexural strength. A significantly higher biaxial flexural strength was shown and decreased in the order of 1 mm, 0.5 mm, and 2 mm specimens (*p* < 0.05). For the firing temperatures groups, a significantly higher biaxial flexural strength was shown, decreasing in the order of 820 °C, 840 °C, 860 °C, 800 °C, and 780 °C groups (*p* < 0.05).

## 4. Discussion

Thermal refinement is an essential process for manufacturing dental restorations composed of LD through the CAD/CAM process. As LD is treated for thermal refinement at temperatures that are able to cause dissolution of LD, the process can affect the optical and mechanical properties of LD. In addition, the thickness of the ceramic is also a major factor that affects the optical and mechanical properties. Therefore, in this study, we aimed to evaluate the translucency and flexural strength of LD glass–ceramics for CAD/CAM at different firing temperatures and thicknesses. To achieve the purpose, translucency measurements and biaxial flexural strength tests were performed on LD specimens of three different thicknesses (0.5, 1.0, 2.0 mm) that were thermally refined at six different firing temperatures (780, 800, 820, 840, and 860 °C). The results showed that a lower translucency was shown in thicker specimens and that the flexural strength decreased in the order of 1.0, 0.5, and 2.0 mm. The translucency and flexural strength were also affected by the thermal refinement temperature. Therefore, the null hypotheses were rejected.

LD is formed through a phase transformation during the heat treatment according to the following equation [18]:

SiO_2 (amorphous)_ + LM _(crystalline)_ = LD _(crystalline)_ (1)

For the present results of the XRD analysis, this study found that the volume fractions of LD increased with an increasing firing temperature. The volume fractions of SiO_2_ increased with an increasing firing temperature from 780 °C to 840 °C but showed a slight decrease at 860 °C. One interpretation of the results might be that β-cristobalite might nucleate at the LM/amorphous matrix interface and then grow at the expense of the amorphous matrix in the first stage. However, LD was nucleated at the LM/β-cristobalite interface and then grew at the expense of LM and β-cristobalite in the second stage [19]. An increase in the primary grain size of LD glass–ceramics was determined with increasing firing temperatures in the present study, which is consistent with reports of other studies that LD crystals can grow from rounded particles with small sizes to elongated rod-like crystals with large sizes because the nucleation sites are limited due to restricted contacts and the reaction between LM and SiO_2_ [20,21].

Regarding the results of the present study on translucency, many previous studies also have shown similar results, showing a decrease in the translucency with an increase in thickness [14,22], which can be explained by the Beer–Lambert law in that a high-thickness material absorbs more lights and results in lower light transmission. When most of the light passing through the ceramic is scattered and diffusely reflected, the material has an opaque appearance [9]. In addition, a previous study also explained that thin material is composed of fewer particles per unit volume than thicker materials and consequently exhibits less scattering and a decreased opacity [23]. According to Table 2, a significant difference in the translucency values was shown between the varying thickness groups, but there was no significant difference between the designed firing temperature groups. The results show that thickness might have a stronger effect on translucency than firing temperatures in LD glass–ceramics for CAD/CAM. The changes in the size of the crystals at different firing temperatures might be so small that the scattering effect of the type and size of crystals could be negligible [24]. Although statistical significance was not observed in Table 2, the results between individual groups shown in Figure 3 showed statistical significance, which was consistent with previous studies [8,24].

In the present study, an interesting result was observed in that a slight decrease in flexural strength occurred after the temperature increased from 820 °C to 860 °C. It was speculated that 780 °C and 800 °C groups with smaller crystals lacked the “interlocking effect” of larger elongated rod-like LD crystals in the 820 °C and 840 °C groups, which could prevent crack propagation, lengthen the path of crack propagation, consume the energy needed for crack propagation, and thus could lead to a higher flexural strength [25]. However, internal residual stresses can arise in LD glass–ceramics upon cooling down from the crystallization temperature due to the mismatch in the coefficients of thermal expansion (CTEs) between the LD phase and the glass matrix, which are 10.1–10.8 × 10^−6^/K and 12.2–12.8 × 10^−6^/K, respectively [26,27,28]. Higher temperatures and larger crystals could cause a higher residual stress, which can produce microcracking, cause severe crystal boundary fractures and crystal dislocations, help crack propagation in glass ceramics, and thus decrease the flexural strength [29,30]. In addition, as the crystallization temperature continued to increase, the crystal growth became abnormal, and irregular crystal particles appeared, which was accompanied by microcracking due to residual stresses, inducing a slight decrease in flexural strength in the 860 °C group [29]. Therefore, the 820 °C and 840 °C groups with medium-sized crystals had both a sufficient “interlocking effect” and a smaller residual stress and exhibited a higher flexural strength than other groups. The flexural strength decreased in the order of 1 mm, 0.5 mm, and 2 mm specimens in the present study, which was consistent with previous studies that observed that increasing the thickness had little effect on the overall flexural strength for each ceramic material when it was beyond a specific thickness [15,16].

The results of this study show that different optical outcomes and flexural strengths of LD glass–ceramics for CAD/CAM can be obtained by modulating the firing temperature in the thermal refinement process and modulating the thickness, which could meet the different requirements for teeth to be restored. For the translucency of ceramics in a clinical situation, it is better to select a more translucent ceramic to restore the teeth without discoloration, but more opaque ceramics are better for teeth with discoloration or metal posts [14]. In general, at least 2 mm-thick LD glass ceramics should be used to mask the effect of the underlying discolored tooth or abutment according to the manufacturer’s instructions [31]. Based on the results of this study, 2 mm LD glass–ceramic specimens with higher firing temperatures could achieve better masking effects, which might be a convenient method to obtain the appropriate esthetic outcomes. The mechanical strength of CAD/CAM lithium disilicate can be different from traditional lithium disilicate in that CAD/CAM lithium disilicate is required to endure mechanical shocks from the milling machine after primary heat treatment and then to endure an additional thermal treatment, which can affect the strength and translucency.

According to the ISO (Internal Organization for Standardization) 6872:2015 standard [17], different clinical indications can be selected based on the different flexural strengths of dental ceramics. In the present study, all the specimens tested were above the threshold of 300 MPa, which meets the ISO requirements for Classes 1, 2, and 3. This result is similar to a previous study in which lithium silicates of both a low translucency and a high translucency requiring thermal treatment were the only materials tested that fulfilled Class 3 ISO 6872:2015 requirements [32]. However, except for the 2 mm specimens at 780 °C (433.49 ± 60.35 MPa) and 800 °C (463.78 ± 96.63 MPa), the other specimens were all above the threshold of 500 MPa, also meeting the ISO requirements for Class 4. However, all the specimens tested were below the threshold of 800 MPa, which does not meet the ISO requirements for Class 5. This means that all the specimens tested are not recommended as a monolithic ceramic for prostheses involving partially or fully covered substructures for four or more units, or for fully covered substructures for prostheses involving four or more units. The results of this study were intended as a reference, as the flexural values reported in ISO 6872:2015 are not related to a specific flexural test. Three flexural test methods are acceptable, including the three-point bending test (3PBT), four-point bending test (4PBT), and biaxial flexural strength test, which may exhibit different results and affect the results [33,34].

Although this study followed international standardization, the used methods lacked some oral environment simulations, such as a humid oral environment, which may lead to the results of this study being different from the results in an intra-oral environment [35]. Moreover, the flat specimens were not clinically anatomic forms, which may also be a limitation of this study. Other flexural test methods and translucency measurement methods may be performed to compare results in further studies. Further studies may be necessary to compare the results and effects of different furnaces, different thicknesses, different compositions, thermocycling, and different thermocycling cycles and temperatures. In addition, this study is a laboratory experiment and the sample size used for this study was referenced from other relevant studies without a power analysis. The sample size is one of the limitations of this study and further experiments strictly following a power analysis will be performed according to the results of this experiment. In addition, further studies to evaluate the mechanical strength of lithium disilicate after setting with various adhesive cements will be valuable.

## 5. Conclusions

The results of this study indicate that as the thickness of lithium disilicate for CAD/CAM and the temperature of thermal refinement decrease, a higher transparency is observed. On the other hand, among thicknesses of 0.5, 1.0, and 2.0 mm, the 1.0 mm-thick samples exhibited the strongest strength, and with 820 °C leading to the highest strength among temperatures of 780, 800, 820, 840, and 860 °C. These findings suggest that it is possible to adjust the transparency and strength according to the clinical situation by selecting an appropriate thickness and an appropriate temperature for thermal refinement.

## Figures and Tables

**Table 1:** Primary grain size (nm) of lithium disilicate glass–ceramics at different firing temperatures (°C).

Temperature	780	800	820	840	860
Primary Grain Size	28.4 ± 5.8	30.0 ± 1.9	32.7 ± 2.8	33.9 ± 1	35.5 ± 4

**Table 2:** Corresponding relationships of thickness and temperature on translucency (T%).

Thickness (mm)	0.5			1		2	
	58.17 ± 1.76 ^c^			39.63 ± 2.37 ^b^		23.43 ± 2.96 ^a^	
**Temperature (°C)**	**780**	**800**	**820**		**840**		**860**
	43.91 ± 13.09 ^a^	39.56 ± 15.19 ^a^	41.02 ± 16.79 ^a^		39.45 ± 14.42 ^a^		38.11 ± 15.77 ^a^

Similar superscript letters indicate homogenous subsets among experimental groups (*p* > 0.05).

**Table 3:** Corresponding relationships of thickness and temperature on biaxial flexural strength (MPa).

Thickness (mm)	0.5			1		2	
	588.17 ± 92.87 ^b^			650.49 ± 118.8 ^c^		508.68 ± 81.66 ^a^	
**Temperature (°C)**	**780**	**800**	**820**		**840**		**860**
	527.37 ± 113.23 ^a^	564.11 ± 134.43 ^ab^	630.24 ± 124.71 ^c^		618.65 ± 100.38 ^c^		571.87 ± 54.06 ^b^

Similar superscript letters indicate homogenous subsets among experimental groups (*p* > 0.05).

## Data Availability

Data are contained within the article.
